# First person – Yarira Ortiz-Alvarado

**DOI:** 10.1242/bio.057448

**Published:** 2020-11-19

## Abstract

First Person is a series of interviews with the first authors of a selection of papers published in Biology Open, helping early-career researchers promote themselves alongside their papers. Yarira Ortiz-Alvarado is first author on ‘Antibiotics in hives and their effects on honeybee physiology and behavioral development’, published in BiO. Yarira is a Research Associate in the lab of Tugrul Giray at the University of Puerto Rico Department of Biology, San Juan, investigating the mechanisms that regulate behavior and development.


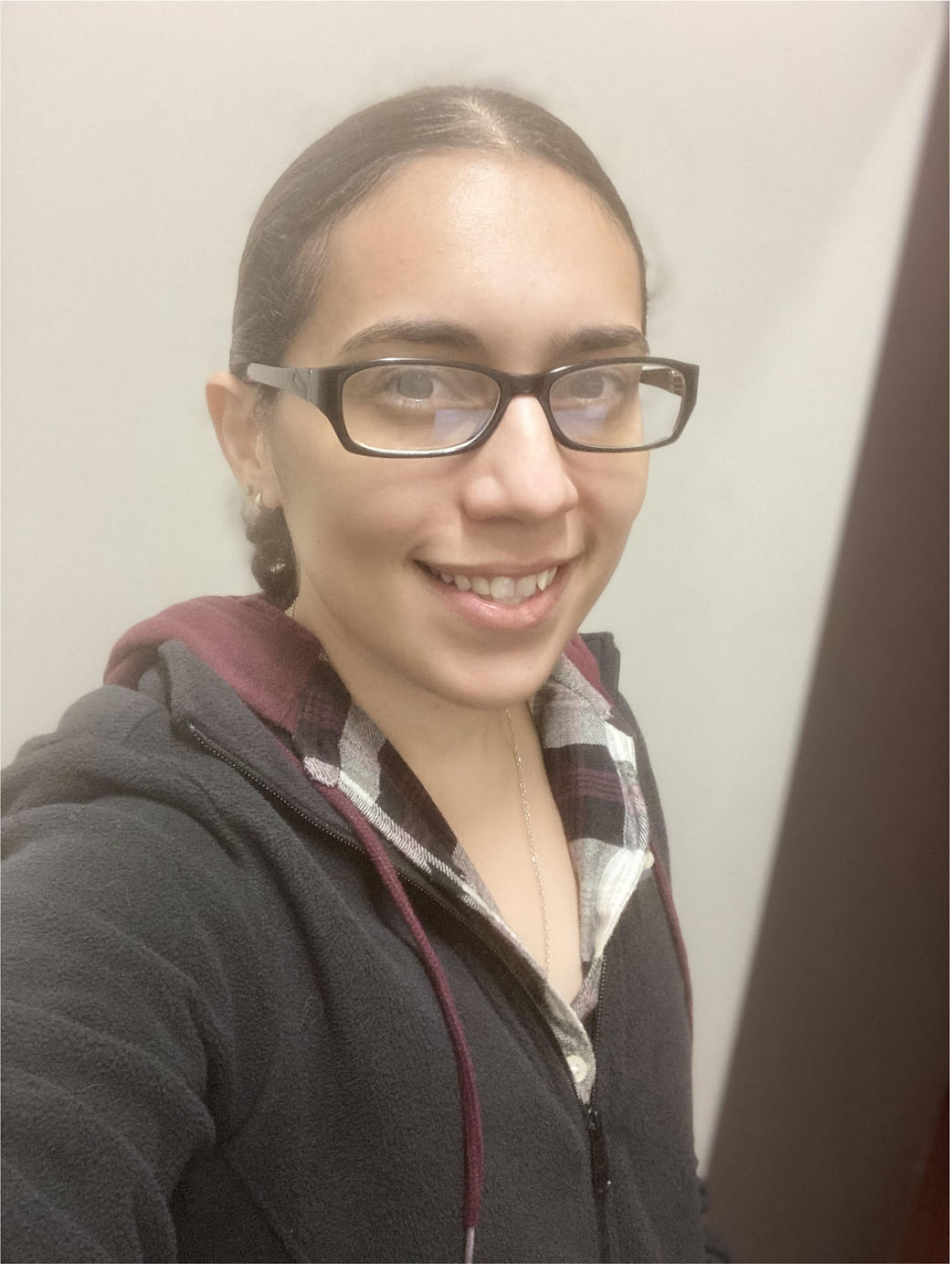


**Yarira Ortiz-Alvarado**

**What is your scientific background and the general focus of your lab?**

My scientific background is on the areas of molecular ecology, development, behavior and gene expression. Our laboratory studies neural, endocrine, and genetic mechanisms of socially relevant behavior from an evolutionary perspective. Our main study organism is one of the pinnacles of social evolution, the Western honeybee. Both mechanistic and evolutionary studies in the lab benefit from a comparative approach across bee (and wasp) species, subspecies, and castes subject to different ecological and evolutionary pressures.

**How would you explain the main findings of your paper to non-scientific family and friends?**

You know how when you get sick the doctor may prescribe you an antibiotic? While you are under treatment, you may notice that you might gain some weight or you're feeling tired or maybe even not hungry at all. In some cases the doctor may even tell you to take a probiotic to make sure the good bacteria in your stomach will get back because the antibiotics will eliminate the good and the bad bacteria. Well honeybees also get sick and given antibiotics. And just like you, they experience changes in their weight and how they behave. It is very likely that just as it happens with us humans, the good bacteria in the honeybee gut changes and as a consequence those physical changes we see in honeybees and even in humans are due to changes in the bacteria.

**What are the potential implications of these results for your field of research?**

The results of this study not only present new insights in the effects of antibiotics on honeybees, but also it may contribute to our understanding of intricate linkage between microbes and development that's important for the host physiology.

**What, in your opinion, are some of the greatest achievements in your field and how has this influenced your research?**

All the way back during my bachelors' research, I wanted to study behaviors in depth, not only induce different outcomes by for example application of hormones, but to understand ‘where’ those different behaviors were coming from. The main part that I considered the big ‘stepping stone’ was the publication of social insects' genomes from honeybees and fire ants. It allowed us the use of different molecular tools to help answer questions such as ‘what gene is related to X behavior? If we knock it down, can the behavior change?’ which has led us to understand the pieces of the mechanisms that drive development and behavior.

**Figure:**
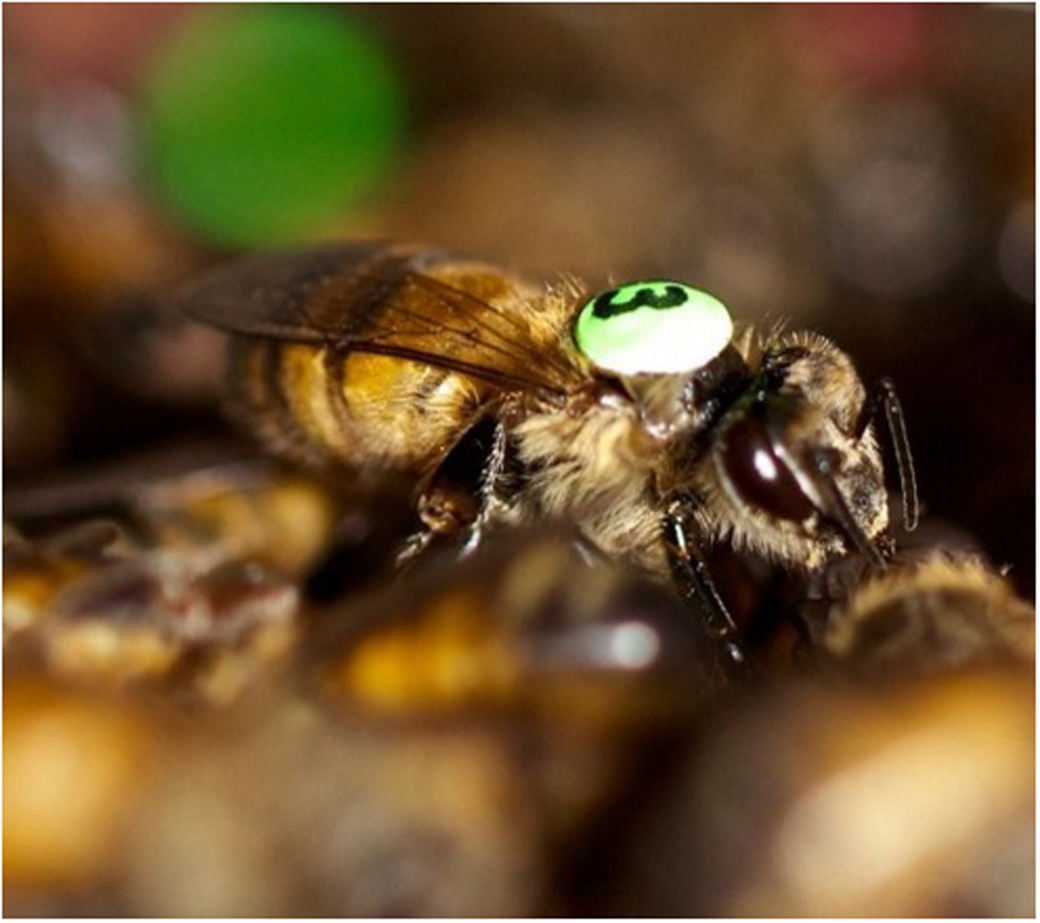
**Tagged honeybee worker.**

“…the big ‘stepping stone’ was the publication of social insects’ genomes from honeybees and fire ants.”

**What's next for you?**

My doctorate research focused on the effect of antibiotics in honeybee microbiota, and how these effects can shape an organism's life history. The importance of microbiota is clear, specifically its role in the interaction between the fat body and the brain of an organism, and behavioral development. I'm interested in studying the role of microbiota in social organisms, which has been found to be correlated with the development of sociality in termites and leaf cutter ants. Microbes may play a significant role in the organization and evolution of social insects and my research hopes to advance our understanding of sociality in insects. As of what's next, I'm currently developing a project that focuses on studying the role of microbiota in regulation of biological clocks during honeybee behavioral development.

“I’m interested in studying the role of microbiota in social organisms…”
